# Inequalities in employment rates among older men and women in Canada, Denmark, Sweden and the UK

**DOI:** 10.1186/s12889-019-6594-7

**Published:** 2019-03-18

**Authors:** Ashley McAllister, Lee Bentley, Henrik Brønnum-Hansen, Natasja Koitzsch Jensen, Lotta Nylen, Ingelise Andersen, Qing Liao, Theo Bodin, Cameron Mustard, Bo Burström

**Affiliations:** 1 Equity and Health Policy Research Group, Department of Public Health Sciences, Karolinska Instiutet, Stockholm, Sweden; 2 Department of Public Health and Policy, University of Liverpool, Liverpool, UK; 3 Department of Public Health, University of Copenhagen, Copenhagen, Denmark; 4 Institute for Work and Health, Toronto, Canada; 5 Unit of Occupational Medicine, Institute of Environmental Medicine, Karolinska Institutet, Stockholm, Sweden; 6 Center for Occupational and Environmental Medicine, Stockholm County Council, Stockholm, Sweden; 7 Dalla Lana School of Public Health, University of Toronto, Toronto, Canada

**Keywords:** Older workers, Employment, Inequalities, Welfare state

## Abstract

**Background:**

In most developed countries, governments are implementing policies encouraging older persons to work past 65 years to reduce the burden on societies related to disability benefits and pension payments. Despite this push to extend working lives, we know little about who already works past this age and any inequalities that may exist. Our study investigates the employment rates of those aged 65–75 years of age by educational level, health status and sex in Canada (CAN), Denmark (DK), Sweden (SE) and the United Kingdom (UK). Secondly, we aim to relate findings on employment rates to prevailing policies in the different countries, to increase the understanding on how to further extend working lives.

**Methods:**

We used nationally representative cross-sectional survey data from the 2012–2013 Canadian Community Health Survey, 2013/14 Survey of Health, Ageing and Retirement in Europe for Denmark and Sweden and the 2013 English Longitudinal Study of Ageing to examine employment rates for those aged 65–75 years by sex, educational level and health status (having limiting longstanding illness (LLI) or not).

**Results:**

Employment rates decline by age, but we see a linear decline in CAN and the UK compared to an initial decline then a plateau of employment rates from 66 to 68 years in DK and SE. Employment rates among persons aged 65–75 years were lower in the UK than in CAN, DK and SE. Among women, employment rates were highest in SE. Women with low education and a LLI had considerably lower employment rates than men with low education and a LLI (employment rates for men ranged from 27% to 12% compared with employment rates for women which ranged from 12% to 0%).

**Conclusions:**

Our results suggest that educational level, sex and health all play a role in extending working lives. The variation in employment rates between the four countries implies that policies do matter, but that social differentials show that policies cannot be ‘one size fits all’. Policy-makers must consider different groups (i.e. low-educated women with a LLI) when designing policies to extend working lives.

## Background

In most countries, rising life expectancy creates opportunities to extend working lives beyond 65 years. Yet little is known regarding who already works past this age and any social differentials that may exist. In the present study, we address this gap by describing employment profiles of ageing workers in four different welfare states–Canada, Denmark, Sweden and the United Kingdom (UK).

### The push to extend working lives

Many countries are developing policies to encourage older workers to remain in paid employment, thus delaying retirement [1], to reduce the burden on societies related to disability benefits or pension payments [2]. In the European Union, the old age dependency ratio (the number of older people as a proportion of those of working age) is expected to rise to 38% by 2030 [3]. Extending working lives may also improve the living conditions of older workers, through higher pension levels. While it is outside the scope of this paper to argue whether employment past 65 years of age is good or bad, we recognise that employment is one factor noted in the public health literature contributing to healthy ageing and a key priority of most governments [4].

Despite the policy push to work past 65 years and older, little evidence on inequalities exists about who remains in employment. We aim to address this gap by identifying inequalities in employment rates among older workers aged 65–75 years focusing mainly on those aged 65–69 years. While several factors may influence the decision to extend ones working life [3], an obvious factor is poor health. Poor health could force a person to exit employment even if they would prefer to continue working [5]. Indeed, evidence shows that health is a key determinant of early retirement [6]. Another important factor is sex. Studies show that, in general, employment rates are lower among women than men [7, 8]. This might indicate greater caring responsibilities for children and older relatives among women than among men. Finally, educational level is another major factor. For example, in Sweden, there are considerable differentials in the length of working life in different occupations. In fact, Nilsson et al. [9] show that workers with lower educational levels tend to leave employment earlier than high educated workers due to health reasons or unemployment. One explanation could be that those with lower levels of education typically have more physically demanding jobs than those with higher levels of education and therefore their work capacity is more affected by decreases in health than those with higher levels of education [10, 11]. Attempts at extending working lives may therefore potentially further aggravate social differentials in employment rates as well as in living conditions. However, a limitation of studies such as Nilsson et al. [9] is that the focus neglects workers older than 65 years. We found only two comparative studies that examine employment rates for those aged 65 years and older [12, 13]. A growing body of literature (see for example [14–16]) about ‘unretirement’ – the process of returning to work after retirement and ‘bridge employment’ (also known as partial employment) – includes our age group of interest but focuses on other aspects such as the meaning of work (see for example [17–19]), life satisfaction (see for example [20]) rather than inequalities. Our contribution to the literature is a comparative, public health focused examination of inequalities in employment rates past age 65 years. To the best of our knowledge, this is the first study to take such an approach.

### Rationale for case selection

This paper is an introduction to a larger project called Tackling Health Inequalities and Extending Working Lives (THRIVE) funded by the Joint Programming Initiative–More Years Better Lives. The THRIVE collaboration builds on previous comparative work that identified inequalities in the general working age population (see for example [7, 21, 22]) between Social Democratic and Liberal welfare regimes. As such, the selection of two Social Democratic welfare regimes–Denmark and Sweden and two Liberal welfare regimes–Canada and the United Kingdom (the UK) are based on these previous collaborations, extending research on employment and health to the older age groups. The following briefly explains the THRIVE theoretical framework.

### The THRIVE theoretical framework

Decisions to retire are multifaceted and structural factors related to policies can influence extending working lives. For example, the design of the pension system (e.g. earlier retirement could lead to lower pension levels), active labour market policies (ALMPs) (e.g. re-training older workers for local labour market demands) and employment protection policies (e.g. anti-discrimination laws, more permanent (and protected) work) [23]. Diderichsen, et al. [24] provides a theoretical framework for analysing how these policies may mitigate the effects of social disadvantage on the risk of disease and poor health and subsequent adverse social and economic consequences at older ages.

**Fig. 1:**
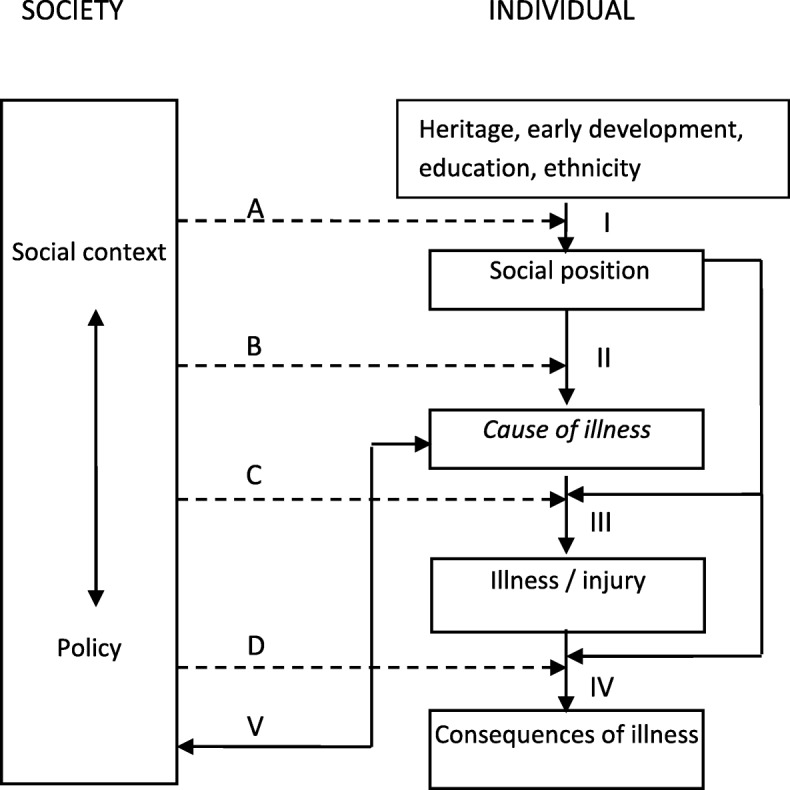
Mechanisms (I − V) and policy entry points (A − D) related to social inequality in health [24]

As illustrated in Fig. 1, policy entry points for intervention may target mechanisms at various levels operating between social disadvantage and limiting longstanding illness [24]. Social stratification (entry point A) involves the allocation of wealth and power in society (e.g. via education) and can impact on the path into social positions. Social protection policies (e.g. social insurance and social assistance) can help alleviate the income-related inequalities that exist due to social stratification. Policies also influence the exposure and the effect of being exposed to risk or protective factors (entry point B and C). For example, work environment policies may protect workers from health-hazardous exposures. Entry point D encompasses policies to mitigate the negative social and economic consequences of the disease (e.g. employment protection legislation, rehabilitation and work training to bring back people to work). For example, Social Democratic welfare regimes in the Nordic countries provide stronger social protection, family policies and employment protection policies than Liberal welfare regimes [25], resulting in higher employment rates among persons with low education and health problems in Sweden compared to the UK [7, 26, 27]. In THRIVE, we hypothesise that policies aimed at increasing employment among the general working age population (i.e. policies in Social Democratic welfare regimes) would facilitate higher employment rates also among older workers. The prevailing level of unemployment in the country could also affect the choices and chances of employment of older persons. In 2013, the unemployment rates in the different countries were 5.9% (CAN), 7.1% (DK), 8.1% (SE), and 7.5% (UK), respectively [28].

### Aims and research questions

The primary aim of this study was to investigate social and health differentials in employment rates among men and women aged 65–75 years and how this compares across Canada, Denmark, Sweden and the UK. Our specific research questions were:

1. How do employment rates vary in the age group 65–75 years and educational level across the four countries?
2. How do employment rates vary among healthy men and women aged 65–69 years by educational level across the four countries?
3. How do employment rates vary among men and women aged 65–69 years with a limiting longstanding illness by educational level across the four countries?

Based on results from the research questions above, we plan to identify which inequalities (e.g. between men and women, different socio-economic groups or specific countries) to focus on in future studies.

### Brief overview of the country policy contexts to extend working lives

#### Canada

Canada’s population of 36 million has had a steadily growing proportion of seniors, increasing from 8% in 1960, to 16% in 2015. In 2015, more than 1 in 6 Canadians were over the age of 64 [29]. As a result of the ageing population, the Canadian Government has implemented numerous policies aimed at extending working lives among older workers [30, 31]. In 2012, changes to the Canadian Pension Plan (CPP) allowed more flexibility for older workers to combine pensions with employment income. The amendments also included an adjustment factor that favours those who delay receiving a retirement pension until after the age of 65 and thus increases incentives for continued working. Other complementary reforms aimed to strengthen the employability of seniors include, for example, Employment Benefits and Support Measures (EBSMs) and the Targeted Initiative for Older Workers (TIOW) for the unemployed living in vulnerable communities.

In addition, Canada has a wide range of social programs designed to alleviate poverty and reduce income inequalities among older adults [32, 33]. These include the Federal Old Age Security (OAS), the income-tested Guaranteed Income Supplement (GIS) and Spouse’s Allowance as well as many special programs (e.g., rental subsidy, property tax relief) offered by provincial/territorial governments. Since all levels of government play a role in social programs, there is some variation in policy approaches across Canada.

#### Denmark

Denmark has a population of 5.8 million and is currently facing increasing challenges with an ageing population. Experts predict that the age dependency ratio will increase from 30% in 2012 to 43% by 2050. Overall, Denmark’s Social Democratic welfare state approach is an effective system that protects against poverty and social exclusion [34]. In Denmark, the municipalities play a crucial role in the implementation of labour and social policies [25]. Denmark takes a ‘flexicurity’ approach–low employment protection but with generous unemployment benefits and strong activation policies [7, 35]. Education and re-training of unemployed workers, especially older workers, to match current labour market demands is an important part of active labour market policies. In the past, the Danish Government provided generous incentives to retire early, but due to the increase in the ageing population, several reforms were introduced aiming to lower early retirement and extend working lives [34]. Reforms include increasing the pension age, financial incentives for remaining longer in employment (e.g. through deferred pension and lump sum payments for people eligible for Voluntary Early Retirement Program (VERP), and finally, educating employers about the value of employing older workers.

#### Sweden

Sweden’s population of 10 million has one of the highest shares of older people in the world. Nearly one in five (19.8% in 2017) is aged over 65 years [36]. Sweden is known for its relatively high levels of welfare spending, and a decentralised system of government, meaning the county and municipal levels are responsible for much decision-making.

Sweden has a long history of active labour market policies, including re-training schemes [33]. However, since the 1990s-economic recession, Sweden has reduced its spending on ALMPs [37]. The economic recession in the early 1990s also contributed to a changing labour market, not least for persons with health problems and low skills. Overall, Sweden has focused on economic incentives to encourage *all people* to keep working irrespective of upstream factors such as health, work environment and social aspects at both work and home. In summary, contemporary Swedish reforms have made it expensive to leave work and profitable to stay in work.

#### UK

The UK has the population of 66 million of whom 18% (1 in 5) are aged 65 or older [38]. This proportion is projected to increase to 21% by 2039 [39]. Welfare spending in the UK is close to the Organisation for Economic Cooperation and Development (OECD) average and is lower than in Denmark and Sweden but higher than Canada [40]. Successive governments have identified the extension of working lives to be a priority and have introduced policies aimed at raising the age of retirement. By 2018, the state pension age for women will increase to 65, bringing it in line with that for men, and is set to increase further by 2028. As well as increasing the age of state pension eligibility, some policies have also sought to make working beyond this point more attractive, by offering incentives including tax benefits and a higher eventual pension based on the amount of time deferred. Over a similar period, there has been an aim of reducing the disability-employment gap [41] primarily by tightening the eligibility criteria, which has been subject to considerable criticism in recent years.

## Methods

### Sample and population

We used nationally representative cross-sectional survey data from the the Canadian Community Health Survey (CCHS), Survey of Health, Ageing and Retirement in Europe (SHARE) in Denmark and Sweden and the English Longitudinal Study of Ageing (ELSA). In this paper, our population of interest are individuals aged 65–75 years.

The CCHS is an ongoing series of cross-sectional health interview surveys administered approximately every 2 years by Statistics Canada, the national statistics agency. Using a multi-staged, stratified sampling frame, the CCHS target population consists of household residents aged 12 and older who are living in private dwellings in all provinces and territories. The survey design features and core content have remained largely unchanged during the series of surveys starting in 2001. We used data from the 2012–2013 survey cycle. For more information on the CCHS see [42].

SHARE is a panel study with bi-annual follow-ups regarding health and socioeconomic conditions (www.share-project.org) (for more information on SHARE see [43]). SHARE data was collected as face-to-face interviews carried out by trained interviewers. We used data from the 5th wave collected in 2013/14. The number of participants (and participation rates) in Denmark and Sweden were 4146 (61.0%) and 4556 (33% for the refreshment sample and 59% for the panel sample), respectively. The numbers of interviewed in the age interval 65–75 were 1295 in Denmark and 1914 in Sweden (see Table 1).

**Table 1:** Information on data sources used by country

Country	Data source	Year used	Sample size Age 65–75
Canada	Canadian Community Health Survey (CCHS)	2012–13	65,000^a^
Denmark	Survey of Health, Ageing and Retirement in Europe (SHARE)	2013 (Wave 5)	1295
Sweden	SHARE	2013 (Wave 5)	1914
United Kingdom (UK)	The English Longitudinal Study of Ageing (ELSA)	2012–13	3500

^a^ total survey n, ages 35–75

ELSA is a nationally representative panel survey of people aged 50 and above in England which began in 2002 (for more information on ELSA [44]). The data used here is taken from the 2013 wave. SHARE is partially harmonised with ELSA to facilitate cross-national analysis. ELSA is a household survey and had a response rate of about 70% for at least one household member at wave 1. Attrition is low between waves with 86% of core sample members responding to wave 6. Refreshment samples are included and elicit a similar response rate to wave 1. Cross-sectional weights are available to correct nonresponse bias and attrition. The UK Data Service manages and licenses the use of the ELSA data.

### Definitions

#### Educational level

Educational level was defined using the International Standard Classification of Education (ISCED) categories and divided into three subcategories: Low (0, 1 and 2), Medium (3 and 4) and High (5 and 6). SHARE uses the ISCED categories to measure education. In ELSA the 3-category education variable is derived from a question asking individuals to indicate which qualifications they have. These represent, less than o-level, o- or a-level or equivalent, and higher than a-level. These correspond to the 3-point ISCED categories. In the CCHS, the low ISCED category (0–2) was defined as 10 years of less of formal schooling, the medium ISCED category was defined as 11–14 years of formal schooling without a post-secondary degree, and the high ISCED category was defined as any post-secondary degree.

#### Employment

We defined employment as working more than 1 h per week in the past week. In ELSA respondents are asked if they were ‘in paid employment’ last week.

#### Limiting longstanding illness

We dichotomised ‘Limiting Longstanding Illness’ (LLI) into persons with a LLI and those without a LLI. The following provides specificities about how each country’s survey asked about a LLI:

- In CCHS, if the respondent answered “sometimes or often” to the question: “Does a long-term physical condition or mental condition or health problem reduce the amount or the kind of activity you can do at home, at work or at school?” then they were categorised as having a LLI.
- In SHARE, the respondents who answered ‘yes’ to whether the person has any long-term health problems, illness, disability or infirmity were cateogorised as having limiting longstanding illness (LLI).
- In ELSA, the measure included two questions: 1) Do you have a longstanding illness or condition after injury? 2) If yes, does this condition limit your work capacity or daily activities? An affirmative answer to these questions was coded as the person having a limiting longstanding illness (LLI).

### Data analysis

We stratified employment rates by sex, educational level and health status (having limiting longstanding illness (LLI) or not). We generated descriptive summary tables for all four countries.

## Results

**Fig. 2:**
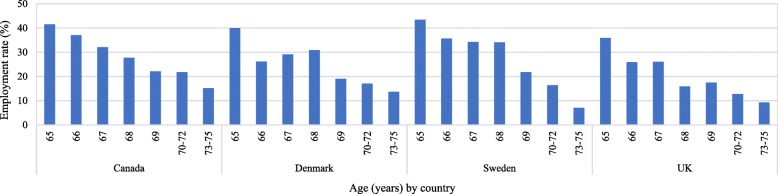
Employment rates among men aged 65–75 by age

Figure 2 provides descriptive results on employment rates among men by age (from 65 to 75 years) in the four countries. Overall employment rates are highest in Sweden and lowest in the UK. Unsurprisingly, Fig. 2 shows that employment rates decline by age. However, the patterns in how the employment rates decline differ across countries. For example, in Canada and the UK, employment rates display a linear pattern of decline from ages 65–75 years. However, in Denmark and Sweden, employment rates plateau from 66 to 68 and drop at age 69 years.

**Fig. 3:**
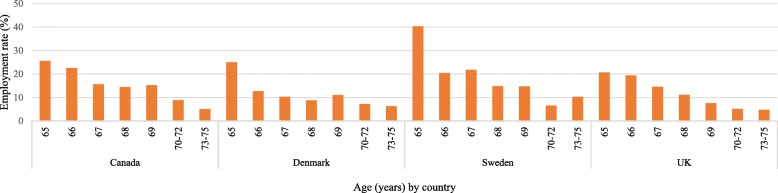
Employment rates among women aged 65–75 by age

Figure 3 shows employment rates among women by age from 65 years to 75 years in the four countries. Employment rates are highest in Canada (except for age 65 years) and lowest in the UK although Denmark and Sweden are not far behind the UK rates. When we compare Figs. 2 and 3, we can see that employment rates are lower among women than men.

**Fig. 4:**
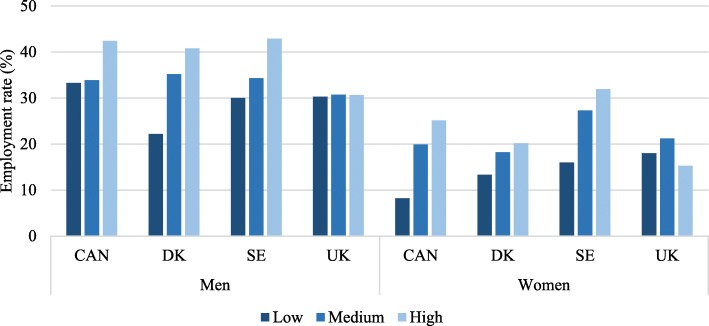
Employment rates of persons without LLI aged 65–69 years by educational level

Figure 4 shows that in all countries and across all educational levels, employment rates are lower among women without LLI than men without LLI, illustrating a gender gradient. Additionally, Fig. 4 shows a social gradient for Canada, Denmark and Sweden since employment rates are highest among those with high education and lowest for those with low education. However, for Canadian men, the social gradient is not as steep as in Denmark and Sweden. For the UK, there is little variation in employment rates by educational level. For men, there is no variation by the educational level at all. The overall pattern shows that employment rates are lowest in the UK when compared to the remaining countries except for low-educated women, where the UK ranks highest and intermediate-educated women where DK ranks lowest. There is a noteworthy variation in employment rates between the countries.

**Fig. 5:**
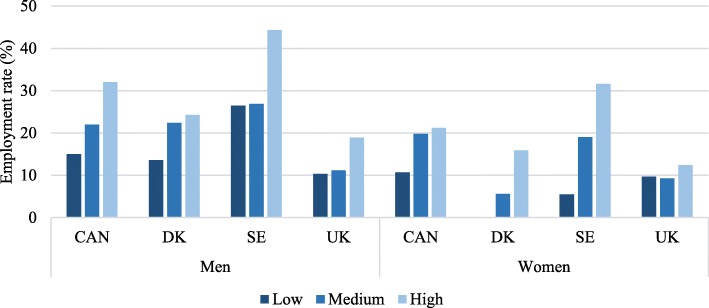
Employment rates of persons with LLI aged 65–69 years by educational level

Figure 5 shows that employment rates are lower for those with LLI than those without a LLI (Fig. 4) showing a health gradient. Overall, we see a much more consistent pattern of social inequality between countries among people with LLI (Fig. 5) than among people without LLI (Fig. 4). However similar to Fig. 4, there is a much smaller social gradient, but with a lower overall employment rate, in the UK than the other countries. There is great variation in employment rates between countries except for low-educated women with LLI, who have low employment rates across all four countries. Also, it is noticeable that the employment rate is higher among highly educated groups for both men and women with LLI. There is little difference between the employment rates among men with and without LLI in Sweden. Among persons with low education and LLI, employment rates are higher among men than among women. Similar to Fig. 4, Fig. 5 employment rates show a social gradient. For highly educated Swedish men and women, employment rates do not vary much between those with and without a LLI. However, having a LLI does seem to be associated with lower employment rates in the remaining three countries.

### 70–75 years

For the age group 70–75 years, stratified employment rates were harder to examine due to the lack of available data on this age group. However, we also calculated employment rates for data that was available in the surveys used for this study by sex, educational level and those with and without a LLI or not for those aged 70–75 years. Similarly, to the age group 65–69, employment rates for this older age group were lower for women (employment rates among men ranged from 27% to 5% compared to employment rates among women ranging from 14% to 1%). For low-educated women with a LLI, employment rates were lower than 5% across all countries.

## Discussion

In this study, we wanted to understand who is working past 65 years of age and if any social differentials exist. Results show that differentials by sex, level of education and health status exist for those that continue working in all four countries. The employment rates among older persons seemed not to be related to the overall unemployment rate in the working population of the country. Sweden had the highest unemployment rate in the population, but among the highest employment rate for persons aged 65–69 years, while the opposite was true for the UK. We can conclude for those that continue working past 65 years in Canada, Denmark, Sweden and the UK, a higher proportion are men, that the employment rate is higher among those with high education than among those with lower education, and that those without a LLI have higher employment rate than those with LLI.

Our results mirror the patterns of inequality in employment outcomes in the literature about working adults under 65 years of age [7, 26, 27]. We contribute to the literature by showing that people do continue working past the age of 65 years. To some extent, our results show similar differentials in employment rates as previous studies on younger adults, regarding sex, educational level and health status. The choice to continue working beyond retirement age is affected by a multitude of factors, and we need better data on why some people continue working and others do not. Results suggest that sex, educational level and having a LLI all play a role. Our results support growing research that there are other important determinants for extending working lives besides financial incentives [45, 46]. Such evidence has important policy implications since most policies focused on extending working life emphasise increasing the financial incentive to work rather than how to accommodate those with poor health.

### Social gradient

Our comparative approach illustrates the variations in who continues to remain in employment. For example, employment rates vary little by educational level in the UK data. For UK men without an LLI the employment rate for low, medium and high educated is 30% in the UK. On the other hand, employment rates are much higher for highly educated persons compared to low-educated persons in Canada, Sweden and Denmark. To illustrate this point, our findings from Sweden show that the employment rate for highly educated women without an LLI is twice as high as the employment rate for low-educated women without an LLI. These results suggest that level of education is a stronger determinant for employment at older ages in Canada, Denmark and Sweden than in the UK. However, this is surprising given that compared to the other three countries, the UK has much more of a class structured society. Our previous studies [47, 48] have shown distinct class patterns in the UK in employment rates among men and women with LLI, compared to in Sweden. Although our present study shows lower overall employment rates in the UK than in Canada and Sweden, more research is needed to understand why there is no (or very little) social gradient among ageing workers in the UK.

### Gender gradient

One explanation for the gender gradient could be that women are more likely to exit paid employment for informal caring responsibilities [49]. Such responsibilities also depend on prevailing policies and arrangements not least concerning the care of older persons. But we expected to see less of a gender gradient in employment rates in Denmark and Sweden given that both countries have strong gender equity policies and provide health and social benefits to alleviate caring obligations that may prevent women from working longer than men. More research is needed to understand the gender gradient in the 65–69-year age group in these settings.

### Health gradient

Our study supports previous research that suggests health plays an important role in continuing to work [50]. While we do not know the exact reasons why those older than 65 years of age are not working, lower employment rates among those with a LLI compared to without a LLI suggest that health could be one factor. However, in this study, we were unable to identify which types of health conditions causing a LLI have higher employment rates than others. Future studies should explore this issue to identify what type of work accommodations are needed for older workers to continue working. Certain work accommodations may be easier to achieve in jobs for high educated persons than for those with low education. Similarly, the needs of persons with physical disabilities may be easier to accommodate than the needs of those with mental disabilities.

### Why cross-country comparison is valuable

Our study provides a comparative perspective on who continues to work longer. Our descriptive analysis illuminates that context matters, aligning with results from Larsen and Pedersen [12]. We propose further questions for consideration by policy-makers when designing policies to extend working life. For example, are ‘flexicurity’ policies–strong social security and weak employment protection–effective at older ages?

Employment rates for Canada were surprising. We expected that employment rates would be more comparable to the UK, another Liberal welfare regime but Canadian employment rates were more comparable to employment rates in Sweden, a Social Democratic welfare regime. A greater understanding of Canada’s approach to extending working lives is needed.

Despite many countries’ push to extend working lives, little research has explored who continues working. Our study contributes to the literature by showing that people are working past 65 years of age but employment rates seem to be influenced by sex, educational level and having a LLI. Better data and reporting on ageing workers is needed to gain insight into why these differentials might exist and policy measures to reduce them. Qualitative interview studies are warranted on how persons reason about their decisions to extend or not extend their working lives, to inform policy making and better guide further quantitative studies.

### Strengths

Only two previous studies [12, 13] focused on the role of health, education and gender on employment rates for those aged 65 years and older across several countries. The social differentials identified in this paper can serve as a starting point for policy-makers and researchers in future policy adaptions and research studies.

While SHARE and ELSA were previously harmonsized, to our knowledge, this is, the first study to include the CCHS to study older persons and employment rates comparatively.

### Limitations

This study is descriptive, so while we can identify differences, we are unable to determine their significance or level of interaction. As such we cannot conclude whether educational differences in employment rates remain after controlling for confounders. Further analytical studies are needed to assess the association between paid work and LLI. Future multivariate analysis should also control for occupation type and income. Also, the sample sizes of the included surveys vary. The number of observations in the SHARE sample used for Sweden and Denmark is much smaller than the other countries, and therefore the estimates may be unstable. A shortcoming of the study is the modest size of the SHARE surveys. The relative high nonresponse rates might introduce a bias because the distribution of educational level among the participants does not reflect that of the population [51]. Combined with differential health status this might imply that illness is more underreported among people with low education than among people with more education. However, if this bias is of similar magnitude in all countries, the cross-country comparison will still be valid. Another limitation is that the wording of survey questions about LLI were phrased differently.

Due to unreliable estimates, employment rates by age do not provide a complete picture. However, our results indicate that people are working past 65 years and that there are social differentials in their employment rates. Future studies need larger sample sizes of older ages collect better employment data for those aged 65 years and older. Also, our study only provides a ‘snapshot’ in time so does not account for ‘unretirement’–the process of retiring and then re-entering work [16]. Platts et al. [16] found that 1 in 4 Britons unretire. However, the authors found that unretirement was more likely among men, persons in good health and those with higher educational attainment, all of which align with our study’s findings.

The definition of work (> 1 h per week in the last week) means that we do not know the intensity of work in different groups or at different ages. It may be that the actual number of hours worked per week varies between groups in ways we have not been able to measure. Further, in the UK, the individuals were asked whether they worked ‘in paid employment’. There might be some misclassification among those who were sick-listed only last week.

Educational attainment is only one way to measure socioeconomic status. Using other indicators (e.g. income or occupation) could have produced different results [52]. We chose educational attainment because this variable was most comparable across datasets.

## Conclusion

Our study shows that those most likely to continue working past 65 years of age are high-educated men without a LLI. Overall, our results demonstrate that social differentials exist for older workers in Canada, Denmark, Sweden and the UK. Older workers are a heterogeneous group, and there are many potential reasons for a person choosing to extend working life such as connectivity, caring responsibilities or financial insecurity. Similarly, there may be obstacles to extending working life, not least social inequalities in health and in workability that could be alleviated through workplace accommodations. Further studies are needed to increase the understanding the drivers of extending working lives, in order to inform policy making.

## Abbreviations

ALMPs: Active labour market policies
CAN: Canada
CCHS: Canadian Community Health Survey
CPP: Canadian Pension Plan
DK: Denmark
EBSMs: Employment Benefits and Support Measures
ELSA: The English Longitudinal Study of Ageing
GIS: The income-tested Guaranteed Income Supplement
ISCED: International standard classification of education
LLI: Limiting Longstanding Illness
OAS: Federal Old Age Security
SE: Sweden
SHARE: Survey of Health, Ageing and Retirement in Europe
TIOW: The Targeted Initiative for Older Workers
UK: United Kingdom
VERP: Voluntary Early Retirement Program
